# Ancestral smoking and developmental outcomes: a review of publications from a population birth cohort^†^

**DOI:** 10.1093/biolre/ioab124

**Published:** 2021-06-25

**Authors:** Jean Golding, Marcus Pembrey, Yasmin Iles-Caven, Sarah Watkins, Matthew Suderman, Kate Northstone

**Affiliations:** Public Health Sciences, Bristol Medical School, University of Bristol, Bristol, UK; Public Health Sciences, Bristol Medical School, University of Bristol, Bristol, UK; Public Health Sciences, Bristol Medical School, University of Bristol, Bristol, UK; Public Health Sciences, Bristol Medical School, University of Bristol, Bristol, UK; Public Health Sciences, Bristol Medical School, University of Bristol, Bristol, UK; Public Health Sciences, Bristol Medical School, University of Bristol, Bristol, UK

**Keywords:** ALSPAC, ancestral childhood smoking, grandmaternal prenatal smoking, child development, sex-specific, non-genetic heredity, obesity, anthropometry, neurocognition, asthma, sensory development

## Abstract

The adverse effects on the child of maternal smoking in pregnancy is well-recognized, but little research has been carried out on the possible non-genetic effects of ancestral smoking prior to the pregnancy including parental initiation of cigarette smoking in their own childhoods or a grandmother smoking during pregnancy. Here, we summarize the studies that have been published mainly using data from the Avon Longitudinal Study of Parents and Children. We demonstrate evidence that ancestral smoking prior to or during pregnancy can often be beneficial for offspring health and both ancestor- and sex-specific. More specifically, we report evidence of (i) adverse effects of the father starting to smoke pre-puberty on his son’s development; (ii) beneficial effects on the grandson if his maternal grandmother had smoked in pregnancy; and (iii) mainly adverse effects on the granddaughter when the paternal grandmother had smoked in pregnancy. The ancestor- and sex-specificity of these results are consistent with earlier studies reporting associations of health and mortality with ancestral food supply in their parents’ and grandparents’ pre-pubertal childhoods.

## Teaser

Review of published ALSPAC studies demonstrate ancestral smoking prior to pregnancy can be beneficial or detrimental for offspring health and development and often both ancestor- and sex-specific.

## Introduction

### Non-genetic heredity

It is widely accepted that plants and animals can adapt to a new environment in ways that can be inherited, but which do not alter the structure of their DNA [1]. The first substantial study to indicate that such effects do occur amongst humans linked details of the health of individuals born on the edge of the Arctic Circle in Sweden to the childhood environments of their grandparents. Studying the years when there was a shortage of food (due to failure of the harvest), and years when there was a glut of food revealed associations with the survival of the grandchildren [2]. Detailed analysis revealed that the associations were dependent on the age and sex of the grandparent when exposed. For example, it showed that when the *paternal* grandfather had been exposed to a particularly lean harvest between ages 9 and 12, his grandchildren lived an average of 15.8 years *longer*; in contrast, if there was an exceptionally good harvest (and thus the 9–12-year-old grandparent had been likely to have overeaten), then the grandchild’s age at death was 16.5 years *lower* [2]. The authors referred to the ages 9–12 as the slow growth period (SGP), but we will use the term pre-adolescence.

Subsequent analyses combined the data with two other cohorts of grandchildren born in 1890 and 1920 in Överkalix. This revealed a sex-specific mortality rate in the grandchildren, such that the mortality rate of the grandsons born in the target years was associated with their paternal grandfathers’ food supply during pre-adolescence and the granddaughters’ mortality rate was associated with the paternal grandmothers’ food supply pre-adolescence [3].

Other studies had taken advantage of the periods of famine in Europe to study the effects of exposure during pregnancy. For example, the period of starvation in parts of Holland in 1944, known as the Dutch Hunger Winter, enabled a study of 37-year-old paternal grandchildren of women who had been exposed during pregnancy. This showed an increase in mean weight and body mass index (BMI) of the grandsons but not the granddaughters. There was no such association in the maternal grandchildren [4].

These studies indicate that when studying the possibility of human non-genetic heredity, it is important to assess the age at exposure of the ancestor, the sex of the exposed ancestor, and the sex of the proband. Here, we describe the studies that have considered the ancestors who smoked, concentrating on the results from the Avon Longitudinal Study of Parents and Children (ALSPAC). The aim of this publication is to identify the epidemiological evidence concerning examples of non-genetic heredity linked to ancestral smoking. We do not, at this stage, think it appropriate to consider causal mechanisms until further evidence is available.

### ALSPAC

This major cohort study was designed overall to determine ways in which different aspects of the environment interact with genes to influence ways in which the child develops into adulthood [5]. It is concerned with details of the lives of the parents (including their childhoods), and the grandparents as well as the children themselves. The study is ongoing. Data have been collected using a number of different sources including: self-completion questionnaires (completed, as appropriate, by parents, teachers, and the probands themselves); direct examination in a clinic setting; linkage to medical, educational and other records; and assays of biological samples. The eligible population comprised the offspring of women who were residents of the county of Avon in south-west England and who were pregnant with an expected date of delivery between 1st April 1991 and 31st December 1992. Approximately 80% of the eligible population took part [6, 7]. The initial number of pregnancies enrolled was 14 541 (for these at least one questionnaire had been returned or a “Children in Focus” clinic had been attended by 19th July 1999). Of these initial pregnancies, there was a total of 14 676 fetuses, resulting in 14 062 live births and 13 988 children who were alive at 1 year of age. Data were collected at various time-points using self-completion questionnaires, biological samples, hands-on measurements, and linkage to other data sets. The study website contains details of all the data that is available through a fully searchable data dictionary and variable search tool: http://www.bristol.ac.uk/alspac/researchers/our-data/.

Ethical approval for the study was obtained from the ALSPAC Ethics and Law Committee (ALEC; ALEC IRB00003312; registered on the Office of Human Research Protections database as UBristol IRB#1) and the three NHS Local Research Ethics Committees (LRECs) that covered the study area (Southmead, Bristol & Weston and Frenchay). ALEC agreed that consent was implied if questionnaires were returned [8]. Further detailed information on the ways in which confidentiality of the cohort is maintained and a full list of ethical approvals may be found on the study website: http://www.bristol.ac.uk/alspac/researchers/research-ethics/.

Among the features that were unique for a birth cohort study at the time were the following: (i) commencing in pregnancy; (ii) consideration of all outcomes, not confined to a single topic; (iii) measures of outcomes were mostly specific to actual measurements or based on traits determined by frequency of signs and symptoms; (iv) signed permission for generic genetic studies [9]; and (v) inclusion of the mothers’ partners (usually the fathers) from the start of the study.

### Cigarette smoking by ALSPAC parents and grandparents

It has been well-established that the individual who smokes is at risk of many adverse health consequences including various types of cancer and coronary heart disease. Less is documented about health associations with parental or grandparental smoking, apart from the implications for exposure of the child to environmental tobacco smoke.

Cigarette smoking is an ideal exposure to investigate retrospectively among parents and grandparents, since it is easily remembered and tends to continue over long periods of time. The ALSPAC study collected information during pregnancy from the study mothers and their partners (F1s) concerning their own smoking histories (including the age at which they started smoking regularly), and current smoking habits. Details were also obtained on the smoking habits of their parents (i.e., the study grandparents F0s), including whether their mothers (the study grandmothers F0s) smoked when pregnant with them. Unfortunately, information on age at which the grandparents started to smoke was not collected at that time; although it has been obtained recently [10] it has not been analyzed at the time of writing (December 2020). Although a direct indication of validation of the historic data is not possible, face validity (i.e., an indication that the association is what would have been expected had the relevant data been available) has shown that the mean birthweights of the mothers (F1s) born to the grandmothers reported to be smoking in pregnancy was substantially lower, in line with expected values if the grandmothers had indeed been smoking [11]. A further confirmation of face validity concerns the answers of the F1 generation to the same questions on the smoking of their mothers in pregnancy (F0s) some 28 years after they were first asked, which showed good consistency (kappa values > 0.43 [10]).

### Age of parents at onset of regular cigarette smoking

As can be seen, very few of the F1 population stated that they had started smoking before age 11 years (1.3% of study mothers and 3.1% of study fathers; Table 1). Analysis of BMI of their offspring (F2) showed that if their fathers (F1s) had started smoking before age 11, their sons but not daughters (F2s) had a higher mean BMI at age 9 [3]. Study of these children until age 17 showed that the excess weight was associated with fat mass and not lean mass [12]. Subsequent analysis of the mean body fat mass of the F2 population at age 24 revealed that the F2s had an excessive degree of fat mass if their parents (F1s) had started smoking early in life, but the susceptible age period differed [13]. For fathers it was <11 years, but for mothers it was between 11 and 15. For offspring of fathers who had started smoking early the excess of fat mass was greatest for sons, whereas for offspring of mothers the excess body fat was greater in the daughters [13].

**Table 1:** Details of smoking habits of ALSPAC parents (F1s) and grandparents (F0s) of the grandchildren (F2s)

Smoking habit	Mother (F1) report		Partner (F1) report	
	*n*	%	*n*	%
F1 ever smoked				
Yes	6701	50.9	5451	55.1
No	6458	49.1	4435	44.9
All	13 159	100.0	9886	100.0
F1 age started regular smoking				
< 11 years	87	1.3	166	3.1
11–12	338	5.1	373	7.0
13–15	1977	29.7	1767	33.0
16	1534	23.2	1108	20.7
>16	2687	40.6	1943	36.2
All known smokers	6623	100.0	5357	100.0
F1 smoked mid-pregnancy				
Yes	2652	20.0	1204	32.8
No	10 622	80.0	6551	67.2
All known	13 274	100.0	9755	100.0
F0 Grandmother ever smoked				
Yes	7194	56.7	5482	56.9
No	5483	43.3	4157	43.1
All known	12 677	100.0	9639	100.0
F0 Grandfather ever smoked				
Yes	9483	76.2	7012	78.6
No	2966	23.8	1909	21.4
All known	12 449	100.0	8921	100.0
F0 Grandmother smoked in pregnancy				
Yes	2956	23.4	1742	18.1
No	7829	62.0	5559	57.9
NK if smoked in pregnancy^a^	1835	14.5	2301	24.0
All	12 620	100.0	9602	100.0

^a^Known to have smoked but not sure whether in pregnancy; these have been included as having smoked in pregnancy.

### Grandmother (F0) smoking in pregnancy

**Figure 1:**
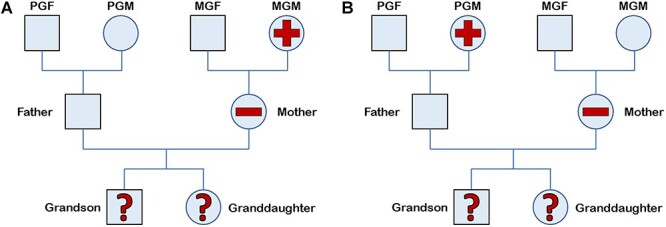
(a) Female-line of inheritance. (b) Male line of inheritance.

We have examined the associations between the grandmother (F0) smoking when the mother (F1) is a non-smoker as illustrated for the maternal and paternal lines in Figure 1a and b. The proportion of F0 grandmothers who smoked in pregnancy was 38.0% for maternal and 42.1% for paternal grandmothers. In comparison, the proportion of F1 mothers smoking mid-pregnancy was 20.0%, mirroring the downward population trend over time (Table 1). A series of publications have so far considered several associations between the grandmother smoking in pregnancy and outcome of the grandchild including: (i) anthropometry, including development of obesity [14]; (ii) neurocognitive traits, including those indicating IQ and autism spectrum disorder (ASD; [15, 16]); (iii) sensory conditions, including aspects of vision and hearing [17, 18]; and (iv) asthma [19]. The results are outlined below.

#### Growth of F2 grandchild

The *fetus* of the smoking mother is well-known to be growth-restricted, with reduction in both birthweight and birth length [20]. A different growth pattern emerged when the grandmother had smoked during pregnancy, but the mother had not. Adjusted analyses showed that the grandsons of the maternal grandmother [MGM] who smoked compared with the grandson of the grandmother who did not smoke weighed more than expected at birth, had a longer birth length, and a greater BMI [14]. There were no such associations for granddaughters, nor for the grandchildren of paternal grandmothers who had smoked in pregnancy [11].

From age 9, the amount of body fat, bone, and lean tissue mass (the lean being mostly muscle) has been assessed and standard measures of height, weight, and waist circumference have been collected regularly. A different growth pattern emerged from that found for fetal growth [14]. If the maternal grandmother had smoked (MGM+), but the mother had not (M−) then, compared with grandchildren of non-smoking mothers and non-smoking maternal grandmothers (MGM − M−), the grandsons had increased weight, BMI, and waist circumference; these measures were shown to be associated with lean but not fat mass. There were no significant associations between MGM smoking and the measurements of the granddaughters.

**Figure 2:**
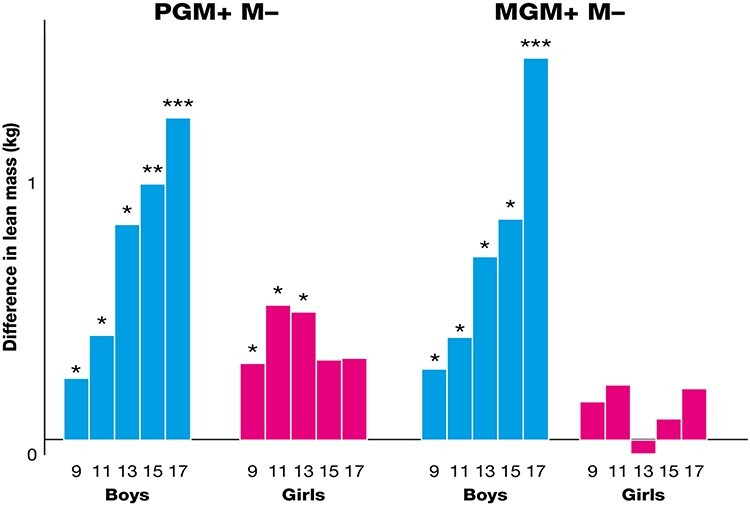
Lean mass of offspring of non-smoking women showing the difference between those whose grandmothers smoked prenatally compared with those who did not (MGM, maternal grandmother; PGM, paternal grandmother; M, mother; + smoked prenatally; − did not smoke prenatally); (^*^) *P* < 0.10; ^*^  *P* < 0.05; ^**^  *P* < 0.01; and ^***^  *P* < 0.001.

A different pattern was found if the paternal grandmother had smoked during pregnancy (PGM + M−). When compared with grandchildren of non-smoking paternal grandmothers (PGM − M−) both grandsons and granddaughters were heavier, had increased BMI, lean, and bone mass; in contrast, the granddaughters but not the grandsons were taller and had increased waist circumference and fat mass. Further analysis of the granddaughters at age 24 confirmed that the granddaughters but not the grandsons had markedly increased adjusted fat mass (interaction between the sexes *P* = 0.001) [13]. The ways in which the lean mass varied with the sex of the grandchild and whether the paternal or maternal grandmother had smoked in pregnancy are illustrated in Figure 2. To determine whether the estimate of lean mass was truly of muscle, we hypothesized that the individuals with excess lean mass would be more likely to be stronger than their peers. As expected, we have shown that this excess lean mass is associated with increased strength [14].

#### Neurocognitive outcomes of the grandchild

To date the ALSPAC data have been examined to assess whether a history of a grandmother smoking is associated with the grandchild’s IQ as measured at ages 8 and 15 [15]. At age 8, IQ was subdivided into its verbal and performance components. After adjustment for the grandmothers’ demographic circumstances there were no associations between IQ and the maternal grandmother smoking in pregnancy. However, there were associations with the paternal grandmother smoking when the mother did not (PGM + M−), such that the grandsons but not the granddaughters had lower mean verbal IQ (mean difference [MD] −2.5 IQ points; 95% confidence interval [CI] −4.4, −0.7). For performance IQ both grandchildren had reduced levels: grandsons MD −2.0 [95%CI −3.6, −0.4]; granddaughters MD −1.8 [95%CI −3.8, −0.1]. There was no association between either grandmother smoking and the IQ of the 15-year old.

An investigation into whether a history of a grandmother smoking prenatally was associated with ASD used four traits (social communication, repetitive behavior, pragmatic communication, and social behavior), high levels of which had been shown to be independently associated with ASD in ALSPAC [21]. Two of these (social communication and repetitive behavior) were associated with the maternal grandmother smoking in pregnancy. The associations were found among the granddaughters, not the grandsons (Table 2). The numbers of grandchildren who had been diagnosed with ASD were relatively small (*n* = 170), with four times as many boys as girls. There was a similar association for each sex with a history of the maternal grandmother smoking prenatally [16].

**Table 2:** The adjusted associations between the highest decile of two autistic traits, and of children with a diagnosis of ASD when the maternal grandmother smoked during pregnancy [MGM + M− v MGM − M−]

ASD characteristic	Grandsons AOR [95% CI]	Granddaughters AOR [95% CI]
Traits associated with ASD		
Social communication	1.04 [0.81, 1.34]	**1.67 [1.25, 2.25]**
Repetitive behavior	1.11 [0.88, 1.41]	**1.48 [1.12, 1.94]**
Diagnosis		
ASD	**1.53 [1.02, 2.29]**	1.56 [0.68, 3.58]

*Notes:* AOR = odds ratio adjusted for grandparents’ demographic characteristics: ASD = autism spectrum disorder; CI = confidence interval; MGM = maternal grandmother; + = smoked in pregnancy; − = did not smoke in pregnancy. Results in bold indicate *P* < 0.05.

#### Vision and hearing

As an example of visual outcomes, we considered myopia (short sightedness) in the grandchildren and showed that the smoking of a grandmother was associated with development of the condition by the age of 7 (but not with myopia that developed after age 7). Unexpectedly, the association was a protective one (Table 3). The children whose grandmother had smoked prenatally were substantially less likely to have developed myopia by this age, particularly if the paternal grandmother had smoked. This association was most dramatic for grandsons, who were 70% less likely than expected to have developed the condition [17].

**Table 3:** The risk of the grandchild developing myopia by age 7 if a grandmother had smoked during pregnancy, but the mother had not

Analysis	Grandsons AOR [95% CI]	Granddaughters AOR [95% CI]
MGM + M− v MGM − M−	**0.52 [0.27, 0.99]**	0.62 [0.34, 1.14]
PGM + M− v PGM − M−	**0.30 [0.13, 0.68]**	0.57 [0.31, 1.05]

*Notes:* AOR = odds ratio adjusted for grandparents’ demographic characteristics CI = confidence interval; MGM = maternal grandmother; PGM = paternal grandmother; + = smoked in pregnancy; − = did not smoke in pregnancy. Results in bold indicate *P* < 0.05.

For auditory outcomes, we considered the children who were particularly sensitive to loud noise since this is a sensitivity that is often associated with autism. Mothers (F1s) were asked about this when their offspring were aged 6 years. The analyses revealed that if the maternal grandmother had smoked in pregnancy then the grandsons were more likely than expected to hate loud noise (adjusted odds ratio [AOR] 1.26 [95% CI 1.00, 1.59]) whereas the granddaughters were less likely to do so (AOR 0.80 [95% CI 0.59, 1.07]). The interaction between these results was significant at *P* < 0.05.

Since the question as to whether the child hated loud noise was subjective, we also analyzed an objective assessment using a recorder and headphones; a tape of music was played that the child was unlikely to have heard before. At age 11, the child was asked to adjust the sound level to one which he/she was comfortable with, and that level was recorded. We found that the grandsons were considerably less likely to set the machine to a high level if their maternal grandmother had smoked (AOR 0.63 [95% CI 0.40, 0.99]), whereas the granddaughters were more likely to set the machine to a high level (AOR 1.64 [95% CI 0.95, 2.83]); once again the sex interaction was statistically significant [18].

#### Asthma and wheezing

ALSPAC had collected detailed information on the frequency with which the child had wheezed, from the first weeks of life throughout childhood. The paper written in 2014 [19] concentrated on three early trajectories based on wheezing frequency between 6 and 42 months (early-onset transitory, early-onset persistent, and late onset), doctor diagnosed asthma and measures of lung function. Adjusted odds ratios showed only two significant associations with grandmother smoking in pregnancy; both involved the paternal grandmother smoking and the granddaughter being at excess risk: persistent wheezing (AOR 1.41 [95% CI 1.09, 1.82]); and doctor diagnosed asthma (AOR 1.39 [95% CI 1.04, 1.86]).

## Discussion

### Age at onset of regular smoking

In this paper, we have summarized the different results that have been described regarding smoking of the grandmothers in pregnancy and of the parents in their own childhoods. For early childhood onset of smoking, we have only considered aspects of growth to date. These have shown that children whose parents started smoking early in childhood were at increased risk of developing excess fat, and that this has continued into early adulthood (age 24). Interestingly, the susceptible ages for onset of smoking differed, being <11 for fathers and 11–15 for mothers; also of note was the fact that fathers’ early onset of regular smoking was associated with excess fat mass of his sons not daughters, whereas the early onset of the mothers’ smoking was associated with excess fat mass of her daughters, not sons [13].

### Smoking of grandmothers in pregnancy

We have shown above, using the ALSPAC resource, that there are many associations linking the grandmother’s smoking in pregnancy with different outcomes in the offspring.

Except for performance IQ, associations tended to always be stronger in one sex of the grandchild as summarized in Table 4. Here, it can be seen that if the association is with the prenatal smoking of the maternal grandmother, her grandsons appear to be the beneficiaries, whether by having increased growth rates, particularly of lean mass and strength, or reduced risk of autistic traits or myopia.

**Table 4:** Summary of sex-specific differences concerning the associations between grandmother smoking during pregnancy and adjusted outcomes to the grandchild (ALSPAC results published as of December 2020)

Grandmother	Grandchild outcome	Grandson	Granddaughter
Maternal (MGM)	Fetal growth	Greater	–
	Lean mass	Greater	–
	Strength and fitness	Greater	–
	Autistic traits	–	Increased
	Myopia by 7	Greatly reduced	Reduced
	Sensitivity to noise	More sensitive	Less sensitive
Paternal (PGM*)*	Height	–	Taller
	Fat mass	–	Increased
	Verbal IQ	Reduced	–
	Persistent wheeze	–	Greater risk
	Diagnosed asthma	–	Greater risk

Conversely when the paternal grandmother smoked in pregnancy, her granddaughters tend to benefit more than the grandsons by being taller and not having a reduced verbal IQ. They were likely to have a greater fat mass in adolescence and early adulthood; although this would be considered a health disadvantage nowadays, in the past this may have been an advantage in regard to successful reproduction.

### Validation of ALSPAC findings

There is only one other study that we are aware of that has considered onset of parental smoking in regard to obesity. Knudsen and colleagues analyzed data from the RHINESSA study and found an association between paternal onset of smoking at age < 15 and fat mass in the sons [22]. Another Nordic study did not find an association between onset of smoking < 11 with BMI of the offspring, but they did not analyze a measure of fat mass [23]; it is of note that Knudson’s analysis also did not find a significant association with BMI, although they did with fat mass. The RHINESSA and other Nordic studies have shown that paternal onset of smoking < 15 years was associated with asthma and reduced lung function in their offspring [24], an association that was also demonstrated in England [25], but the ALSPAC data have not been analyzed in this regard yet.

There are surprisingly few studies that have considered associations with grandmothers’ smoking in pregnancy, especially for paternal grandmother smoking. Nevertheless, there are studies that validate our findings. For example, Svanes and colleagues have reported an association between paternal grandmother smoking and asthma in the grandchild [26], and several studies have demonstrated an increase in birthweight if the maternal grandmother, but not the mother, had smoked in pregnancy [27–29] similar to our own findings [11].

Alternative ways of validating the findings include the use of biomarkers, the most commonly used being DNA methylation. There is consistent evidence that the offspring of a smoking mother has specific DNA methylation markers that differ from those found in blood of individuals who are themselves smokers [30]. We have now shown evidence of DNA methylation biomarkers of grandmother smoking that are again specific to the grandmother who smoked and are frequently sex-specific; interestingly CpG sites on the X chromosome were most likely to be involved [31]. These contrasts are similar in complexity to the sex-specific and grandmother-specific differences we have demonstrated with several of the phenotypes reported here.

## In conclusion

Several papers have now been published using the ALSPAC data that show that pre-adolescent onset of regular smoking of cigarettes of parents and exposure to a grandmother smoking throughout pregnancy have intergenerational effects that are reflected in differing patterns of DNA methylation. We should point out that many of the findings reported here would benefit from being replicated in other data sets. It is also true that the phenotypes considered to date are far from being comprehensive. Most have been chosen for analysis on the basis that they represent conditions which have been increasing in prevalence over several decades (e.g., obesity, autism, and myopia). Other conditions warrant consideration in the future, and subsequent generations should also be considered in association with biomarkers such as DNA methylation.
